# The complete mitochondrial genome of deep-sea ophiuroid *Ophioleila elegans* (Echinodermata: Ophiuroidea) from the Shkolnik Guyot, a northwest Pacific seamount

**DOI:** 10.1080/23802359.2023.2288441

**Published:** 2023-12-06

**Authors:** Chailinn Park, Eun-Bi Kim, Se-Jong Ju

**Affiliations:** a Ocean Georesources Research Department, Korea Institute of Ocean Science and Technology, Busan, Republic of Korea; b Ocean Science Major, University of Science and Technology, Daejeon, Republic of Korea; c Marine Resources & Environment Research Division, Korea Institute of Ocean Science and Technology, Busan, Republic of Korea

**Keywords:** Deep-sea Ophiuroidea, *Ophioleila elegans*, seamount, mitochondrial genome, phylogeny

## Abstract

Ophiuroids are a diversified benthic taxon in the deep sea. Given their various dispersal strategies, they are considered an adequate group to assess genetic connectivity, especially in the seamounts that function as islands. *Ophioleila elegans* A.H. Clark, 1949, in the family Ophiothamnidae, was previously reported from the Caiwei Guyot, a seamount in the northwest Pacific Ocean. Here, we described the mitochondrial genome of *O. elegans* collected from another seamount in the northwest Pacific. The whole mitogenome is 16,376 bp in length and encodes 13 protein-coding genes, two ribosomal RNA genes, and 22 transfer RNA genes. Phylogenetic analysis based on the mitogenome sequences showed that *O. elegans* was clustered with *Histampica* sp., the only species for which mitogenome sequence has been reported within the family Ophiothamnidae. The complete mitogenome of *O. elegans* first reported in the present study provides useful information for population genetics and evolutionary relationship of this taxon, especially in the northwest Pacific seamounts.

## Introduction

Ophiuroids are a particularly represented taxon in most deep-sea benthic habitats and have various strategies for dispersal (O'Hara 2007). Given their abundance and diverse dispersal strategies, they are considered an adequate group to assess genetic connectivity dynamics in seamounts that serve as centers of endemism and stepping stones for dispersal (Cho and Shank 2010; Na et al. 2021). *Ophioleila elegans* A.H. Clark, 1949 is a rarely reported ophiuroid species distributed from the north-central Pacific Ocean to the northwest Pacific seamounts (Zhang et al. 2018). In this respect, *O. elegans* seems to be the appropriate species for evaluating genetic connectivity of seamounts, especially in the northwest Pacific Ocean. Here, we described the complete mitochondrial genome of *O. elegans* and analyzed its phylogenetic relationship with mitogenomes of other ophiuroid species.

## Materials

**Figure 1.**
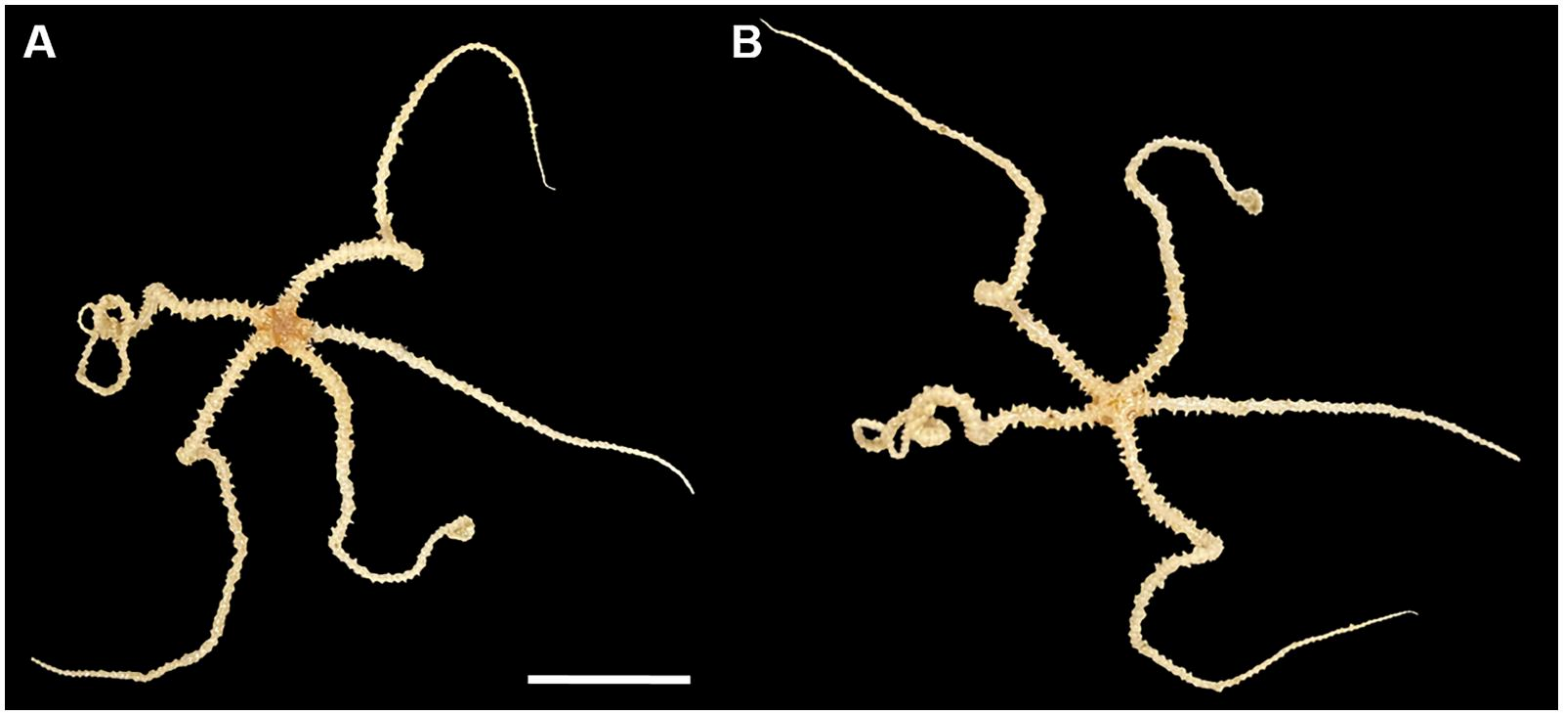
Photographs of the *Ophioleila elegans* (scale bar: 1 cm) collected from the Shkolnik Guyot in the northwest Pacific Ocean (16° 5′ 45″ N, 151° 53′ 6.34″ E; depth: 1474 m). A: aboral view; B: oral view.

During the oceanographic cruise WP21 of the Korea Institute of Ocean Science and Technology (KIOST) in May 2021, the ophiuroid specimens were collected from the Shkolnik Guyot, northwest Pacific Ocean (16° 5′ 45″ N, 151° 53′ 6.34″ E; depth: 1474 m) using ROV URI-L (Redone Technologies, Jangseong, Republic of Korea). Photographs of the specimens were taken on-board with a Nikon DSLR (Figure 1). The specimens were identified as *Ophioleila elegans* by the following morphological characteristics: oral shields small and triangular, adoral shields enlarged trapezoid shape, five arms more than five times the disc diameter (Zhang et al. 2018). The *O. elegans* specimens were deposited at the Marine Biodiversity Institute of Korea (https://www.mabik.re.kr/html/en/, Seok-Chun Ko, and seokchunk@mabik.re.kr) under the voucher number MABIK BK20220511.

## Methods

Genomic DNA was extracted from the specimen using DNeasy Blood & Tissue Kit (Qiagen, Hilden, Germany). The mitochondrial *cox1* region of the specimen was confirmed, which showed 100% sequence identity to *O. elegans* (accession number: KU895364). For the mitochondrial genome analysis, mitochondrial DNA amplification was performed using REPLI-g Mitochondrial DNA kit (Qiagen, Hilden, Germany). The REPLI-g kit was used to generate high yields of enriched mitochondrial DNA, which has already been applied to ophiuroids (Lee et al. 2019). Sequencing libraries were prepared using the TruSeq Nano DNA High Throughput Library Prep Kit (Illumina, San Diego, CA). Genomic DNA of 100 ng was sheared using adaptive focused acoustic technology (Covaris, Woburn, MA) and the fragmented DNA was end-repaired to create 5′-phosphorylated, blunt-ended dsDNA molecules. After end-repair, the DNA was size-selected using a bead-based method. These DNA fragments go through single ‘A’ base addition and ligation to the TruSeq DNA UD Indexing adapters. The products are then purified and enriched with PCR to generate the final DNA library. The paired end (2 × 150 bp) sequencing was performed through Illumina NovaSeq platform (Illumina, San Diego, CA). The low-quality reads and adapter sequences were trimmed by Trimmomatic v0.36 (Bolger et al. 2014), and the trimmed reads were further assembled using SPAdes 3.15.0 (Bankevich et al. 2012). The overlapping region was cut out, the remaining sequence was considered circular, and oriented rotation was performed. The closing of the circle was verified by Sanger sequencing. The assembled genome was annotated using MITOS2 (Donath et al. 2019) and these annotations were manually adjusted by comparison with other ophiuroid mitogenomes. The mitochondrial genome map and the sequencing depth and coverage map were described using the Proksee (Grant et al. 2023) and CircularMapper (Peltzer et al. 2016; Fig. S1), respectively. Since the genus *Ophioleila* is monotypic, and only one mitogenome sequence (*Histampica* sp. Am Clark, 1970; MZ442339) has been reported within the family Ophiothamnidae, eight mitogenome sequences belonging to the suborder Gnathophiurina were added for phylogenetic analysis, together with three outgroup species (Table 1). We aligned the 13 protein-coding genes (PCGs) using MAFFT v7.49 (Katoh and Standley 2013) and selected the evolutionary best-substitution model for each gene through IQ-TREE webserver (Trifinopoulos et al. 2016). A phylogenetic tree was reconstructed by the maximum-likelihood (ML) method with the 1000 bootstrap replicates and the Bayesian inference (BI) method using RAxML-NG v1.2.0 and MrBayes v3.2.7, respectively (Ronquist et al. 2012; Kozlov et al. 2019). The bootstrap support (BS) values from the ML and the Bayesian posterior probability (BPP) from the BI were indicated in each node. The phylogenetic tree based on the ML algorithm was visualized using FigTree v1.4.4 (http://tree.bio.ed.ac.uk/software/figtree).

**Table 1.** Mitochondrial genomes belonging to the suborder Gnathophiurina and three outgroup species used for phylogenetic analysis.

Species	Family	Accession no.	Reference
*Ophioleila elegans* A.H. Clark, 1949	Ophiothamnidae	OQ207673	This study
*Histampica* sp. Am Clark, 1970	Ophiothamnidae	MZ442339	Li et al. (2021)
*Amphioplus laevis* (Lyman, 1874)	Amphiuridae	MN276320	Xu et al. (2019)
*Amphiura digitula* (H.L. Clark, 1911)	Amphiuridae	MK343096	Lee et al. (2019)
*Amphiura sinicola* Matsumoto, 1941	Amphiuridae	MK343094	Lee et al. (2019)
*Ophiactis savignyi* (Müller & Troschel, 1842)	Ophiactidae	MZ268820	Unpublished
*Ophiopholis aculeata* (Linnaeus, 1767)	Ophiopholidae	AF314589	Smith et al. (1993)
*Ophiopholis japonica* Lyman, 1879	Ophiopholidae	MK343095	Lee et al. (2019)
*Ophiopholis mirabilis* (Duncan, 1879)	Ophiopholidae	MK343098	Lee et al. (2019)
*Astriclypeus mannii* Verrill, 1867 (outgroup)	Astriclypeidae	OL502703	Shin et al. (2022)
*Florometra serratissima* (AH Clark, 1907) (outgroup)	Antedonidae	AF049132	Scouras and Smith (2001)
*Holothuria forskali* Delle Chiaje, 1824 (outgroup)	Holothuriidae	FN562582	Perseke et al. (2010)

## Results

**Figure 2.**
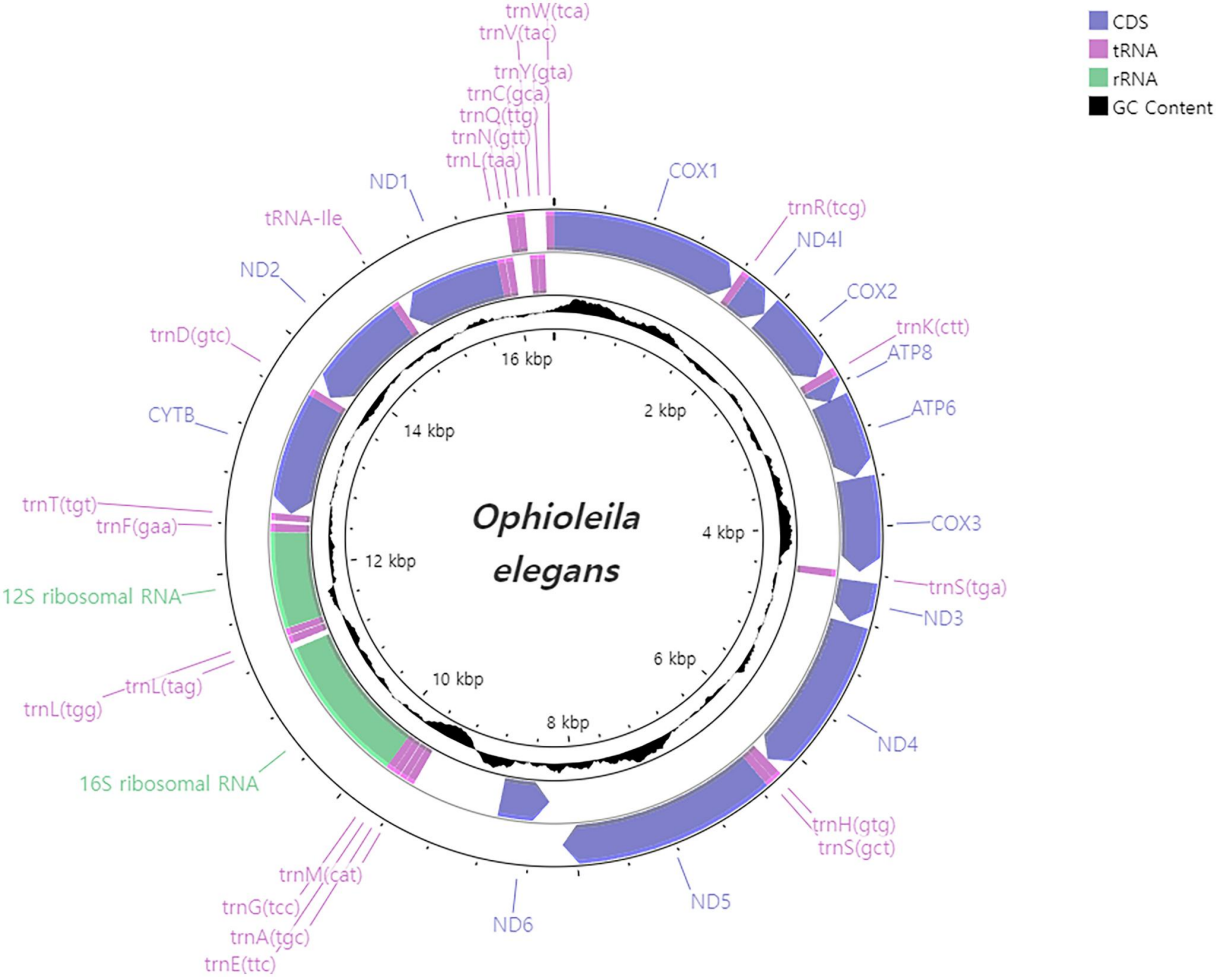
Mitochondrial genome map of *Ophioleila elegans*. GC content was plotted by default values of window size (500) and score scale (1).

**Figure 3.**
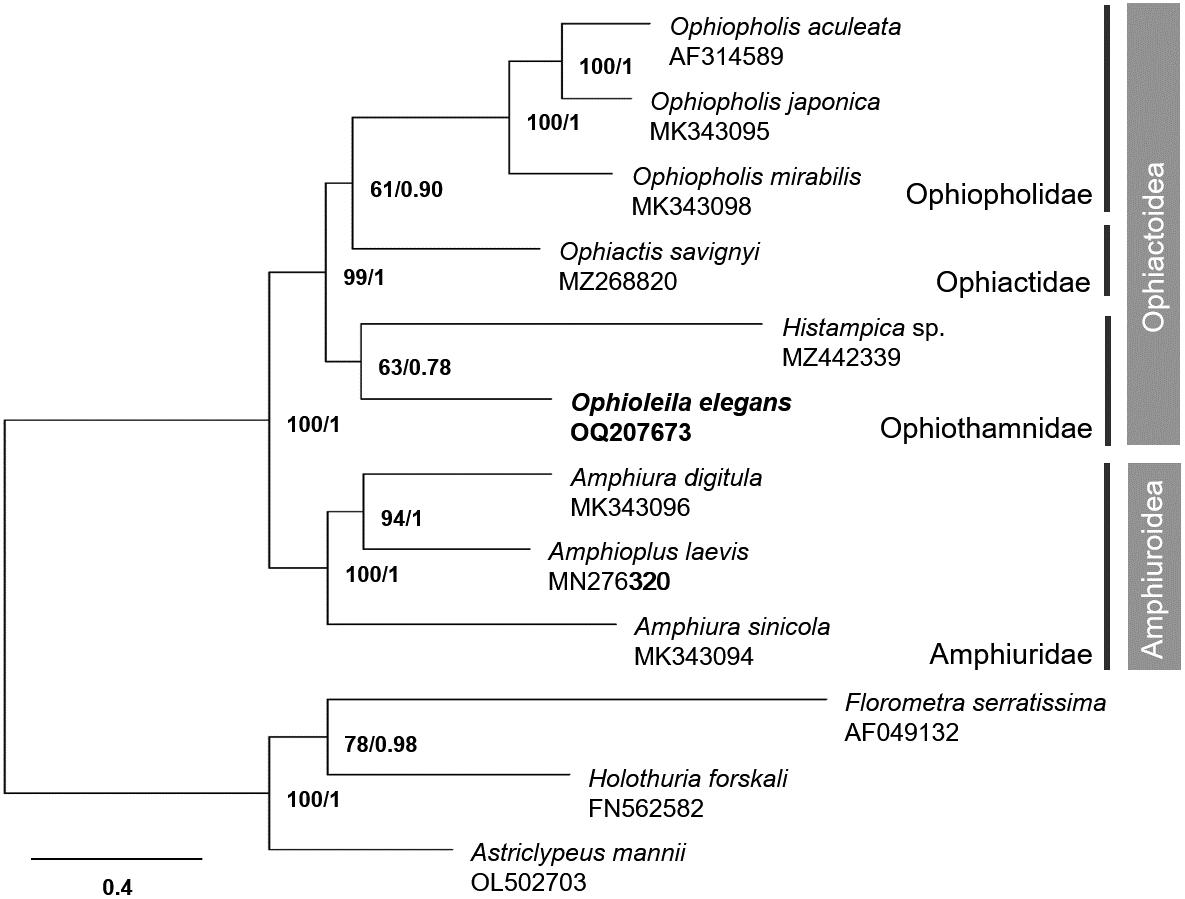
The maximum-likelihood (ML) phylogenetic tree of *Ophioleila elegans* and other ophiuroid species based on 13 protein-coding mitochondrial genes. Numbers on each node denote the bootstrap support values from the ML and the Bayesian posterior probabilities from the Bayesian inference (BI) method. The scale bar indicates the number of substitutions per site.

The whole mitogenome of *O. elegans* is 16,376 bp in length (GenBank accession no. OQ207673), with 64% and 36% of AT and GC contents, respectively (Figure 2). It consists of 13 PCGs, two ribosomal RNA genes (rRNAs), and 22 transfer RNA genes (tRNAs). All PCGs have ATG as the start codon and TAA as the stop codon, except *cytb* and *cox1*, which start with GTG. Phylogenetic analysis based on 13 PCGs shows that *O. elegans* is most closely related to *H*. sp. (MZ442339), but with weak support (BS: 63, BPP: 0.78; Figure 3).

## Discussion and conclusions

The conserved tRNA cluster (Asn-Gln-Cys-Val-Tyr-Trp) commonly identified in the order Amphilepidida, to which *O. elegans* belongs (Lee et al. 2019), was also found in this study. In the phylogenetic analysis, *O. elegans* and *H*. sp. were clustered with low BS and BPP values. *Ophioleila* was tentatively placed in the family Ophiothamnidae with the genera *Ophiothamnus* and *Histampica* based on the exon-capture phylogeny (O'Hara et al. 2017; Zhang et al. 2018). However, the low support value in the phylogenetic analysis between *O. elegans* and *Ophiothamnus* species has also been reported in previous study (Zhang et al. 2018). Therefore, the additional mitogenome data should be supplemented to better understand the phylogenetic relationships of the family Ophiothamnidae. The complete mitogenome of *O. elegans* first reported in the present study would provide helpful information for future studies on evolutionary relationships of this deep-sea taxon.

## Supplementary Material

Supplemental Material

Supplemental Material

## Data Availability

The mitogenome sequence data that support the findings of this study are openly available in GenBank of NCBI at https://www.ncbi.nlm.nih.gov/ under the accession no. OQ207673. The associated BioProject, SRA, and Bio-Sample numbers are PRJNA943565, SRR23824997, and SAMN33731640, respectively.
