# Supplementary figures and images for: The complete mitochondrial genome of deep-sea ophiuroid Ophioleila elegans (Echinodermata: Ophiuroidea) from the Shkolnik Guyot, a northwest Pacific seamount
Source: Mitochondrial DNA B Resour. 2023 Dec 6;8(12):1334–8. doi: 10.1080/23802359.2023.2288441 (PMC10768936; doi:10.1080/23802359.2023.2288441)

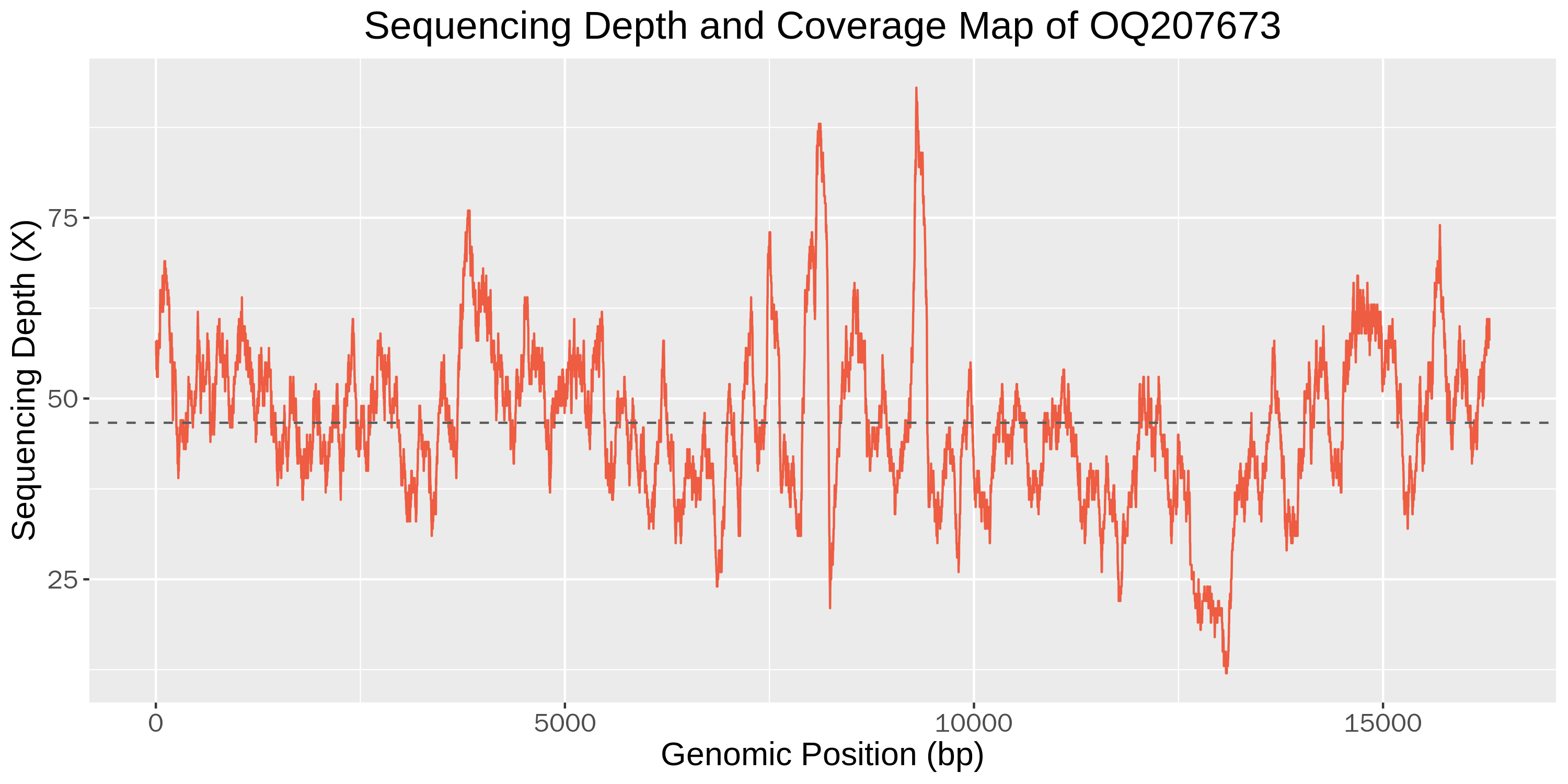

Supplement: Supplemental Material [file TMDN_A_2288441_SM2731.png]
